# Structural Optimization Design of the Dual-Layer CMUT with Low Power Consumption and High Ultrasonic Reception Performance

**DOI:** 10.3390/mi16070782

**Published:** 2025-06-30

**Authors:** Jie Li, Zhaohui Xiao, Zutang Wu, Xiong Hu, Zhikang Li, Yihe Zhao, Min Li, Jiawei Yuan, Shaohui Qin, Libo Zhao

**Affiliations:** 1 The College of Mechanical and Electrical Engineering, Shaanxi University of Science and Technology, Xi’an 710021, China; lijie@sust.edu.cn (J.L.); 18174221326@163.com (Z.X.); 2 Basic Education College, National University of Defense Technology, Changsha 410073, China; huxiong18@nudt.edu.cn; 3 Northwest Institute of Nuclear Technology, Xi’an 710024, China; wuzutang@163.com; 4 State Key Laboratory for Manufacturing Systems Engineering, State Industry-Education Integration Center for Medical Innovations, International Joint Laboratory for Micro/Nano Manufacturing and Measurement Technologies, Shaanxi Innovation Center for Special Sensing and Testing Technology in Extreme Environments, Shaanxi Provincial University Engineering Research Center for Micro/Nano Acoustic Devices and Intelligent Systems, Xi’an Jiaotong University, Xi’an 710049, China; yihezhao@xjtu.edu.cn (Y.Z.); limin@xjtu.edu.cn (M.L.); yjw19971106@stu.xjtu.edu.cn (J.Y.); 3121301031@stu.xjtu.edu.cn (S.Q.); 5 School of Instrument Science and Technology, Xi’an Jiaotong University, Xi’an 710049, China

**Keywords:** low power consumption, high ultrasonic reception performance, dual-layer CMUT

## Abstract

Capacitive micromachined ultrasonic transducers (CMUTs) have been widely applied in fields such as air-coupled ultrasonic nondestructive testing, gesture recognition, and 3D imaging. However, most current CMUTs struggle to simultaneously achieve both low power consumption and high performance, which limits their application in relevant fields. In this paper, a dual-layer CMUT is proposed, and its structural optimization design is also analyzed. The dual-layer CMUT consists of a top-layer circular CMUT cell and a bottom-layer annular CMUT cell. A movable pillar connects the top and bottom cells of the double-layer CMUT. This design increases the total deflection and reduces the stiffness, making the membrane more susceptible to deformation under external forces, thereby achieving low power consumption and high reception performance. The finite element method (FEM) results showed that, compared with conventional CMUTs, the structural optimization design of the dual-layer CMUT had a 13.7% reduction in collapse voltage. The improvements in the maximum deflection, average deflection, electromechanical coupling coefficient, transmitting sensitivity, and receiving sensitivity were 41.2%, 68.0%, 84.6%, 17.7%, and 101.6%, respectively. Therefore, the dual-layer CMUT has low power consumption and high reception performance while maintaining transmission performance, and it has potential for applications in portable, low-power devices and air-coupled ultrasonic nondestructive testing.

## 1. Introduction

Ultrasonic technology is a detection and imaging method based on the physical properties of sound waves. In recent years, with advances in microelectromechanical system (MEMS) technology and the rapid development of the Internet of Things (IoT), ultrasonic technology has made significant progress in various fields, including medical imaging and therapy [1,2], industrial nondestructive testing [3], biometric fingerprint recognition [4], and contactless operation and actuation [5,6]. Ultrasonic transducers are the core components of ultrasonic technology applications, significantly influencing the detection accuracy, imaging quality, and functionality of the entire ultrasonic system. Therefore, continuous innovation and development of ultrasonic transducers are important.

The capacitive micromachined ultrasonic transducer (CMUT) is an ultrasonic transducer based on MEMS technology, which has the advantages of wide bandwidth, high electromechanical coupling coefficient, and good acoustic impedance matched with human tissue and facile integration with integrated circuits [7,8,9]. Compared with conventional piezoelectric ultrasound transducers [10], CMUTs have demonstrated significant advantages in fields such as air-coupled ultrasonic nondestructive testing [11,12], biochemical sensing [13,14], and medical imaging [15,16]. However, as the requirements for low-power, portable, and highly integrated applications of ultrasonic transducers continue to increase in various technological fields [17,18], how to reduce the operating voltage of CMUTs and how to maximize the performance of CMUTs have become urgent areas for improvement.

G. G. Yaralioglu et al. [19] showed that the higher the electromechanical coupling coefficient, the higher the electromechanical conversion efficiency when the bias voltage of the CMUT was close to the collapse voltage. Therefore, the higher loaded collapse voltage ratio of the CMUT (generally a maximum of 80–90% of the collapse voltage ratio), the better performance of the CMUT. Therefore, the CMUT needs to reduce the collapse voltage so that it can have a high electromechanical coupling coefficient at low operating voltages. To address this, A. Kshirsagar et al. [20] designed a dielectric layer CMUT structure using Si_3_N_4_ material as a vibrating membrane using the electrode-charging method. After the Si3N4 material is charged, it can trap a certain amount of charge, thereby generating an electric potential difference. Once the Si_3_N_4_ material is pre-charged, a predefined electric field is formed, which reduces the required loaded bias voltage. C. Seok et al. [21] reduced the power consumption of CMUTs by employing an intermittent power supply strategy through circuit design. In recent years. S. Bang et al. [22] have reduced the collapse voltage of CMUTs by replacing the SiO_2_ insulating layer with a high-k (relative permittivity) insulating layer. This approach ensures the reliability of CMUT silicon wafer bonding while lowering the collapse voltage. C. Goel et al. [23] used a novel rocker support stem to hold the vibrating membrane in order to reduce the effective stiffness of the CMUT, so the CMUT could generate a larger deflection for the same electrostatic force, which achieved the effect of reducing the collapse voltage.

With the increase in vibration speed, amplitude, and area, the output sound pressure of the vibrating circular disk gradually increases [24]. However, the presence of the CMUT cavity limits the amplitude of the CMUT vibrating membrane, which affects the performance of the CMUT. At the same time, CMUTs operate in a bending vibration mode, which has a smaller average amplitude compared with piston-like vibration, further limiting the performance of CMUTs. Therefore, in order to improve the performance of CMUTs, the average amplitude of the membrane is usually increased by increasing the cavity height or causing the membrane vibration pattern to tend towards piston-like vibration in order to achieving the improvement in its performance. To address this, Y. Huang et al. [25] improved the output sound pressure of a CMUT cell by adding a mass block to the center of the membrane structure of a conventional CMUT cell, thus creating a non-uniformly vibrating membrane. This design made the vibration mode of the membrane more similar to the piston-like vibration, which resulted in a more homogeneous surface deflection and improved the output sound pressure of the CMUT cell. This design provided ~100% improvement in transmitting sensitivity and receiving sensitivity compared with conventional CMUTs. R. O. Guldiken et al. [26] proposed a non-homogeneous thickness, multi-electrode vibrating membrane structure in which the center electrode was loaded with direct current (DC) bias voltage and the external annular electrode was loaded with alternating current (AC) excitation voltage. This design ultimately resulted in a high electromechanical coupling coefficient of 0.82 at 8 MHz center frequency by integrating a non-homogeneous-thickness membrane structure with a center mass block. D. K Kim et al. [27] proposed an indirectly clamped CMUT; this configuration allowed the bottom plate to vibrate in a piston-like manner, resulting in a 149% increase in the electromechanical coupling coefficient and a 72% improvement in the output sound pressure. S. Na et al. [28] proposed a CMUT cell structure with an annular vibrating membrane. Compared with the conventional circular vibrating membrane, the ratio of the average deflection to the maximum deflection of the annular vibrating membrane was larger, which led to a larger vibrational deflection, and the measured transmitting sensitivities were 33.83 Pa/V and 25.85 Pa/V when this CMUT was excited by a continuous wave and a 20-cycle burst, respectively, achieving the goal of enhanced ultrasonic output power. Z. Li et al. [29] introduced a novel annular electrode CMUT structure, in which the design of the annular electrode made the CMUT tend more towards a piston-like vibration during bending vibration. This configuration improved the average deflection, acoustic pressure, and electromechanical coupling coefficient of the CMUT.

Although the above studies made significant progress towards reducing the power consumption and improving the performance of CMUTs, most of the studies struggled to achieve both low power consumption and high performance in CMUTs. In this paper, a dual-layer CMUT (D-CMUT) with both low power consumption and high reception performance is proposed. After its structural optimization design process, the research results showed that, compared with the conventional CMUT, the D-CMUT had low power consumption and high reception performance while maintaining transmission performance.

## 2. Design and Concept of D-CMUT

**Figure 1:**
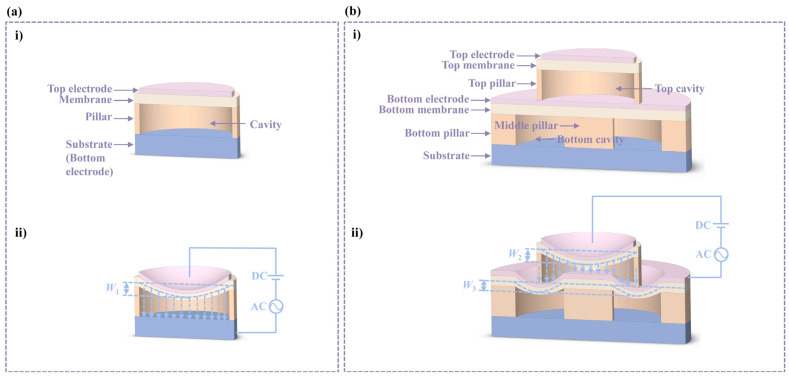
Schematic diagram of D-CMUT and comparison with conventional CMUT. (**a**) Conventional CMUT: (**i**) structural diagram and (**ii**) membrane deflection under electrostatic force. (**b**) D-CMUT: (**i**) structural diagram and (**ii**) membrane deflection under electrostatic force.

A schematic diagram of the D-CMUT and comparison with a conventional CMUT are shown in Figure 1. The D-CMUT consists of top and bottom cells, the top cell is a circular CMUT and the bottom cell is an annular CMUT, where the top circular CMUT cell has the same dimensions as the conventional CMUT structure.

The pillar of a conventional CMUT is fixed and immovable. However, the top pillar of the D-CMUT is movable. When DC bias voltage is loaded, the top membrane deforms under electrostatic force, while the force generated at the top pillar deforms the bottom membrane. Therefore, the superposition of its own deflection and the deflection of the top pillar can increase the total electrostatic deflection, prompting the ultrasonic transducer to collapse at lower voltages, achieving the effect of low collapse voltage and low power consumption without changing the height of the cavity. This design also allows the top membrane to deform more under the same external force, resulting in higher reception performance.

**Figure 2:**
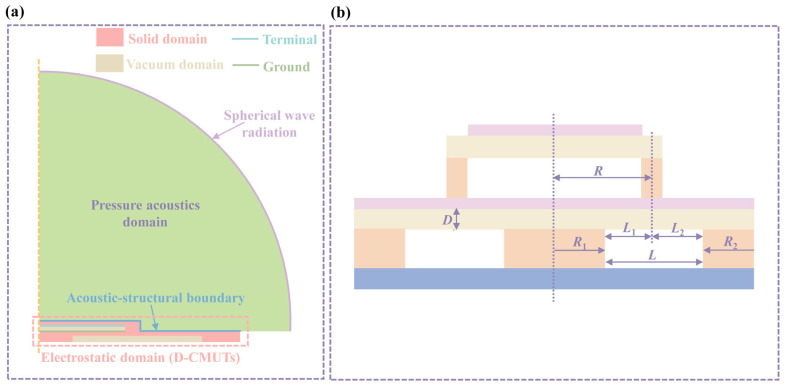
FEM and structural parameter diagram. (**a**) 2D axisymmetric electromechanical–acoustic multiphysics coupling model diagram and (**b**) structural parameter diagram.

In order to analyze the basic performance of the D-CMUT, a finite element model (FEM) of the D-CMUT was built by COMSOL 6.1a. The model contains an electromechanical module (electrostatic domain) and an acoustic module (pressure acoustics domain). In Figure 2a, the electrostatic domain consists of a solid region (light red region) and a vacuum region (light brown region), forming an electromechanical multiphysics coupling model that is commonly used to analyze collapse voltages, capacitances, and other performance characteristics of D-CMUTs. In this model, terminals (light blue lines) and ground (dark green lines) are located at the ends of the vacuum region of the top cell. In addition, dynamic meshing is used for all vacuum regions, while electromechanical multiphysics fields are used for all solid regions. To ensure that the top pillar can be moved, only the middle and bottom pillar are fixed. It is worth noting that the substrate was neglected in the simulation process. This design, in the modeling process, was intended to improve simulation efficiency. In the pressure acoustics domain, spherical wave radiation is located at the outer boundary of the physical field, while the acoustic–structure boundary is located between the solid domain and the pressure acoustics domain. Finally, the electrostatic domain and the pressure acoustic domain together form an electromechanical–acoustic multiphysics coupling model for analyzing the acoustic pressure, transmitting sensitivity, receiving sensitivity, and other performance characteristics of the D-CMUT.

The structural parameters of the D-CMUT are shown in Figure 2b, and all the structural parameters and material properties used for FEM are illustrated in Table 1. In the structural parameter diagram (Figure 2b), *R* = 50 µm.

## 3. Results

### 3.1. Collapse Voltage

**Figure 3:**
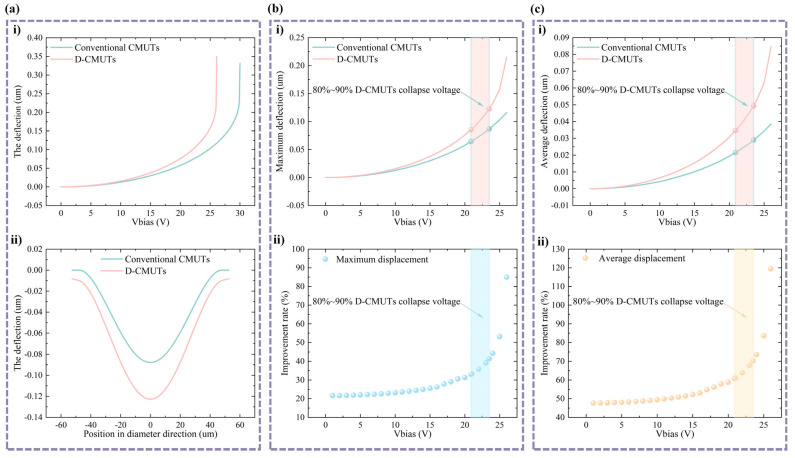
Maximum and average deflections of two CMUTs at different bias voltages. (**a**) Collapse voltage and membrane shape: (**i**) collapse voltage and (**ii**) membrane shape. (**b**) Maximum membrane deflection analysis: (**i**) maximum membrane deflection at different bias voltages and (**ii**) maximum membrane deflection improvement rate. (**c**) Average membrane deflection analysis: (**i**) average membrane deflection at different bias voltages and (**ii**) average membrane deflection improvement rate.

The collapse voltage is an important characteristic of CMUTs. The collapse voltage determines the power consumption of the CMUT, the lower the collapse voltage, the lower the power consumption of this CMUT. In Figure 3a(i), as the DC bias voltage increases, the deflection at the center of both types of CMUT also increases. When the DC bias voltage load is 26.1 V, the deflection at the center of the D-CMUT increases dramatically, indicating that the collapse voltage of the D-CMUTs is 26.1 V. However, in the conventional CMUT, the center point deflection increases significantly only when the bias voltage reaches 30.0 V. Therefore, the collapse voltage of the D-CMUT is lower than that of the conventional CMUT. The collapse voltage of the D-CMUT is reduced by 13.0% compared with that of the conventional CMUT. In addition, the reduction rate of collapse voltage can be further improved by adjusting the structural dimensions of the CMUT.

### 3.2. Membrane Deflection

The membrane deflection magnitude usually affects the performance of CMUTs and is one of the key parameters. The maximum and average deflections of the conventional CMUT and the D-CMUT under different DC bias voltages were calculated by static simulation analysis. In Figure 3a(ii), it can be seen that when both the conventional CMUT and the D-CMUT are loaded with 90% of the D-CMUT collapse voltage (23.5 V), the deflections of the points on the D-CMUT membrane are larger than the deflections of the corresponding points on the conventional CMUT membrane. This is due to the deflection of the top pillar of the D-CMUT. Considering that the normal operating voltage of a CMUT is 80–90% of its collapse voltage, the analysis focused on the case where both the conventional CMUT and the D-CMUT were loaded to 80–90% of the D-CMUT collapse voltage (20.9 V–23.5 V). In Figure 3b, when the loaded DC bias voltage is 80–90% of the D-CMUT collapse voltage, the maximum deflections of the D-CMUT are increased by 33.1–41.4% compared with the conventional CMUT. From the average membrane deflection analysis (Figure 3c), it can be seen that the average deflection of the D-CMUT increased by 60.8–70.3% under the same conditions.

### 3.3. Electromechanical Coupling Coefficient

**Figure 4:**
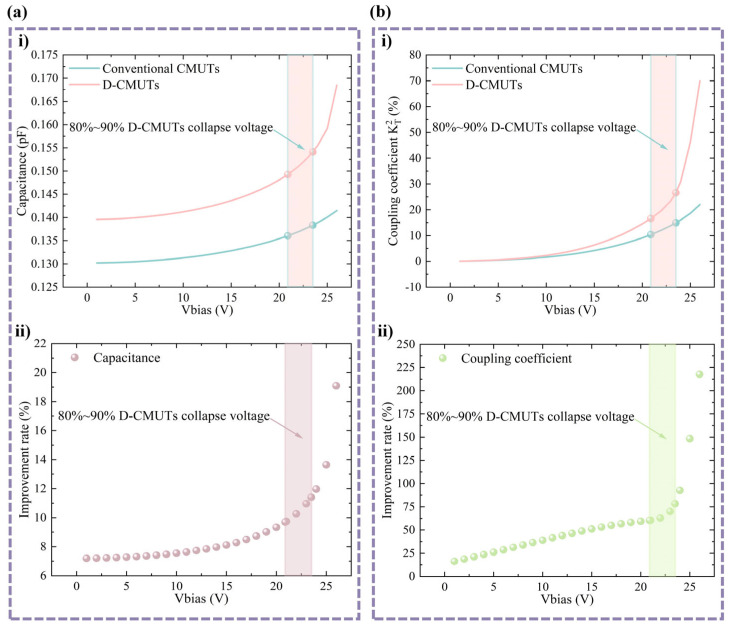
Capacitance and electromechanical coupling coefficients of two CMUTs at different bias voltages. (**a**) Capacitance analysis: (**i**) capacitance at different bias voltages and (**ii**) capacitance improvement rate. (**b**) Electromechanical coupling coefficient analysis: (**i**) electromechanical coupling coefficient at different bias voltages and (**ii**) electromechanical coupling coefficient improvement rate.

Before analyzing the electromechanical coupling coefficients, parametric scanning was performed to obtain the capacitance at different bias voltages based on the static analysis. The capacitance of two CMUTs at different bias voltages is shown in Figure 4a. The capacitance of the D-CMUT is higher than that of the conventional CMUT under the same loaded DC bias voltage, which facilitates the testing of the CMUT signal. In addition, at the same DC bias voltage, the slope of the capacitance–voltage curve of the D-CMUT is significantly larger than that of the conventional CMUT.

In Figure 4b, the electromechanical coupling coefficients of the D-CMUT at different bias DC voltages can be expressed by the method proposed by G. G. Yaralioglu et al. [19,30]. Based on the known capacitance, the electromechanical coupling coefficient can be calculated with expression [31].

(1) $$K_{T}^{2}=1-\frac{C^{S}}{C^{T}}$$

Here, $K_{T}^{2}$ represents the electromechanical coupling coefficient, *C^S^* is the fixed capacitance, and the slope of the charge–voltage curve is usually defined as the free capacitance *C^T^*:

(2) $$C^{S}=\frac{Q(w)}{V_{bias}}{|}_{{w,V}_{bias}}{=C(w)|}_{{w,V}_{bias}}$$

(3) $$C^{T}=\frac{dQ(w)}{{dV}_{bias}}{|}_{{w,V}_{bias}}{=C(w)|}_{{w,V}_{bias}}{+V}_{bias}\frac{dC(w)}{{dV}_{bias}}{|}_{{w,V}_{bias}}$$

where *V_bias_* represents the loaded DC bias voltage.

The discrete capacitance data *C*(*w*) given in Figure 4a are fitted using an exponential function to replace *C^S^*. The exponential functions and fitting parameters of D-CMUTs with typical structures are shown in Table 2.

Based on the above equation, the electromechanical coupling coefficients of the D-CMUT and the conventional CMUT at different bias voltages are drawn (Figure 4b(i)). In Figure 4b(ii), the electromechanical coupling efficiency of the D-CMUT is improved by 60.4% to 78.3% compared with that of the conventional CMUT when the voltages from 20.9 V to 23.5 V are loaded simultaneously.

### 3.4. Ultrasonic Transmitting and Receiving Performances

**Figure 5:**
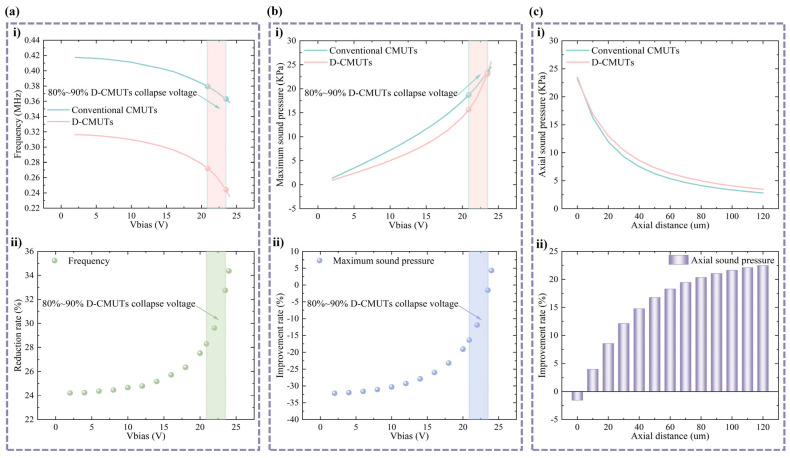
Frequency and sound pressure of two CMUTs at different bias voltages. (**a**) Frequency analysis: (**i**) frequency at different bias voltages and (**ii**) frequency reduction rate. (**b**) Maximum sound pressure analysis: (**i**) maximum sound pressure at different bias voltages and (**ii**) maximum sound pressure improvement rate. (**c**) Center axial distance sound pressure analysis: (**i**) sound pressure at center axial distance and (**ii**) sound pressure improvement rate.

The frequency results of two CMUTs are shown in Figure 5a. It can be seen that the frequency of the D-CMUT is lower than that of the conventional CMUT for the same bias voltage because the unfixed top pillar generates deflection, resulting in lower stiffness. When both CMUTs are loaded to bias voltages ranging from 20.9 V to 23.5 V, the frequency reduction of the D-CMUT is 28.3% to 32.7% compared with the conventional CMUT.

The maximum sound pressures of the conventional CMUT and D-CMUT at different bias voltages were calculated by frequency domain analysis. In Figure 5b, although the maximum sound pressure of the D-CMUT is lower than that of the conventional CMUT at low bias voltages, the maximum sound pressure of the D-CMUT has a higher rate of change with increasing bias voltage. The normal operating voltage of the CMUT is 80% to 90% of the collapse voltage. When both the conventional CMUT and D-CMUT are loaded to DC bias voltages ranging from 20.9 V to 23.5 V and 0.5 V AC excitation voltage, the difference in maximum sound pressure between the D-CMUT and the conventional CMUT is relatively small. Analyzing the axial sound pressure at the center point when the DC bias voltage is 23.5 V and the AC excitation voltage is 0.5 V (Figure 5c), the sound pressure of the CMUT decreases with the increase in the axial distance. The lower frequency of the D-CMUT has less attenuation in the medium than a conventional CMUT, so that the sound pressure is higher at the farther center points. Additionally, in applications such as air-coupled nondestructive testing and non-contact operation and control, non-near-field sound pressure is typically used. Therefore, D-CMUTs have certain advantages in terms of sound pressure performance.

**Figure 6:**
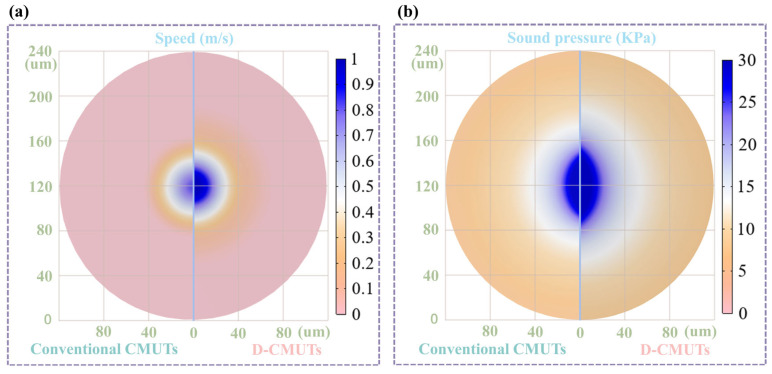
Distribution diagram. (**a**) Speed field distribution of conventional CMUTs and D-CMUTs. (**b**) Sound field distribution of conventional CMUTs and D-CMUTs.

In Figure 6, the movable top pillar in the D-CMUT connects the top cell with the bottom cell, causing them to vibrate and emit ultrasonic waves. The superposition of these two waves maintains the ultrasonic transmission performance. The sound field distributions of the conventional CMUT and the D-CMUT are shown in Figure 6b. Under the same medium conditions, the amplitude of the sound pressure is proportional to the frequency and vibration speed [32]. When both 23.5 V DC bias voltage and 0.5 V AC excitation voltage are loaded, the frequency of D-CMUT is smaller than that of the conventional CMUT (Figure 5a), but the vibration speed of the D-CMUT is higher than that of conventional CMUT (Figure 6a). Therefore, the maximum sound pressure of both CMUTs is essentially the same (Figure 6b).

**Figure 7:**
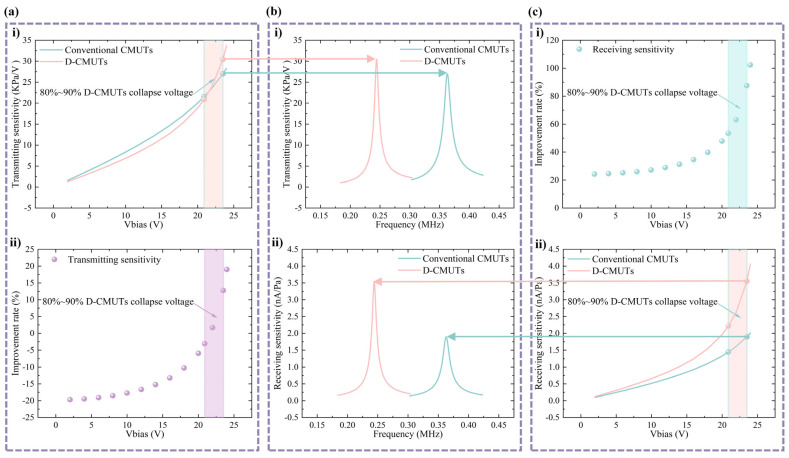
Transmitting and receiving and sensitivity of two CMUTs at different bias voltages. (**a**) Transmitting and sensitivity analysis: (**i**) transmitting sensitivity at different bias voltages and (**ii**) transmitting sensitivity improvement rate. (**b**) Sensitivity: (**i**) transmitting sensitivity and (**ii**) receiving sensitivity. (**c**) Receiving sensitivity analysis: (**i**) receiving sensitivity improvement rate and (**ii**) receiving sensitivity at different bias voltages.

Ultrasonic transmitting sensitivity and receiving sensitivity are key parameters for evaluating the performance of CMUTs. These parameters affect the effectiveness and quality of CMUTs in applications such as air-coupled nondestructive testing, ultrasound imaging, and ultrasound therapy. The transmitting and receiving sensitivities of two CMUTs at different bias voltages are shown in Figure 7. The transmitting and receiving sensitivities of two CMUTs were studied by harmonic interference under cell excitation (1 V AC voltage or 1 Pa sound pressure) at a certain DC bias voltage [29]. The transmitting sensitivity is defined as the average absolute sound pressure at the top membrane surface at the resonance frequency, while the receiving sensitivity is defined as the value of the generated current. In Figure 7a, the rate of improvement in the transmitting sensitivity of the D-CMUT is −3.1% to 12.8% compared with the conventional CMUT when the DC bias voltage of both CMUTs is 80% to 90% of the collapse voltage of the D-CMUT. The receiving sensitivities of the two CMUTs are shown in Figure 7c. When the loaded DC bias voltage is 80% to 90% of the D-CMUT collapse voltage, the receiving sensitivity of the D-CMUT is improved by 53.4% to 87.5% compared with the conventional CMUT. The larger the mean signal, the higher the signal-to-noise ratio (SNR), the larger the mean noise, and the lower the SNR [33]. As shown in Figure 7c, the current signal of the D-CMUT is significantly larger than that of the conventional CMUT under the same external force. However, the noise usually comes mainly from electrical noise, and the D-CMUT is less affected by the heterogeneous structure compared with the conventional CMUT. Therefore, the SNR of the D-CMUT is higher than that of the conventional CMUT.

## 4. Structural Optimization Design and Discussion

### 4.1. Structural Optimization Design

In a D-CMUT, the top pillar is the key component that connects the top circular CMUT cell and the bottom annular CMUT cell. The vibration of the bottom annular CMUT cell needs to be driven by the top pillar. At the same time, the stiffness of the top pillar has a significant effect on the vibration of the top circular CMUT cell. Therefore, the design of the bottom membrane thickness (*D*), the position of the top pillar acting on the bottom cavity (*L*_1_/*L*), and the bottom cavity width ratio (*L*/*R*) are optimized. (It is worth noting that the normal DC bias operating voltage for CMUTs is typically 80% to 90% of the collapse voltage. Therefore, during the optimization design process, a DC bias voltage of 90% of the D-CMUT’s collapse voltage and an AC excitation voltage of 0.5 V are loaded).

**Figure 8:**
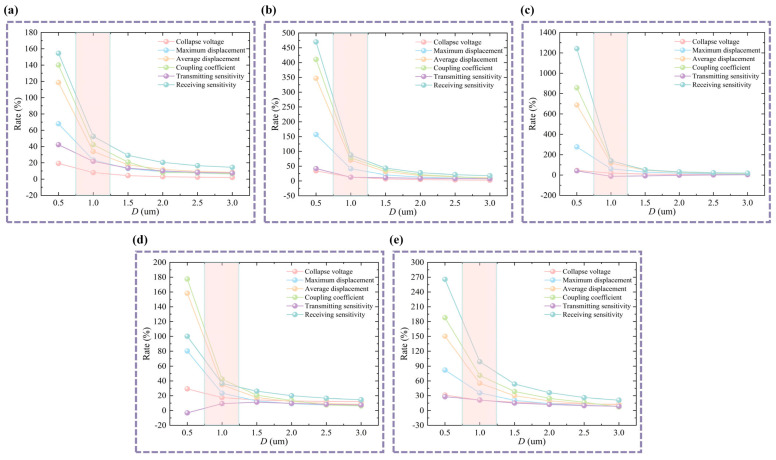
Analysis of variation in bottom membrane thickness of D-CMUTs. (**a**) Analysis of variation in bottom membrane thickness *D* under *L*/*R* = 60% and *L*_1_/*L* = 50%. (**b**) Analysis of variation in bottom membrane thickness *D* under *L*/*R* = 100% and *L*_1_/*L* = 50%. (**c**) Analysis of variation in bottom membrane thickness *D* under *L*/*R* = 140% and *L*_1_/*L* = 50%. (**d**) Analysis of variation in bottom membrane thickness *D* under *L*/*R* = 100% and *L*_1_/*L* = 30%. (**e**) Analysis of variation in bottom membrane thickness *D* under *L*/*R* = 100% and *L*_1_/*L* = 70%.

The stiffness of the top pillar and the deflection of the bottom membrane depend, to some extent, on the bottom membrane thickness. Therefore, analyzing the bottom membrane thickness (*D*) is a key step in the optimized design of the D-CMUT structure. The design was optimized by adjusting the bottom membrane thickness while keeping the bottom cavity width ratio (*L*/*R*), the position of the top pillar acting on the bottom cavity (*L*_1_/*L*), and other dimensional parameters unchanged. The top circular CMUT cell always matches the structural dimensions of a conventional CMUT. The results are shown in Figure 8. Most of the performance rates (collapse voltage is reduction rate) decrease gradually as the *D* value increases. Although there is no stabilizing trend for the transmitting sensitivity rate, the range of variation in transmitting sensitivity is relatively small compared with other performance parameters. Therefore, it is preferable to choose a thinner bottom membrane thickness. However, given current manufacturing capabilities, the bottom membrane thickness (*D*) is 1 µm (corresponding to the rectangular color area). Naturally, with the continuous improvement in manufacturing technology, choosing a thinner bottom membrane will significantly enhance the performance of D-CMUTs.

**Figure 9:**
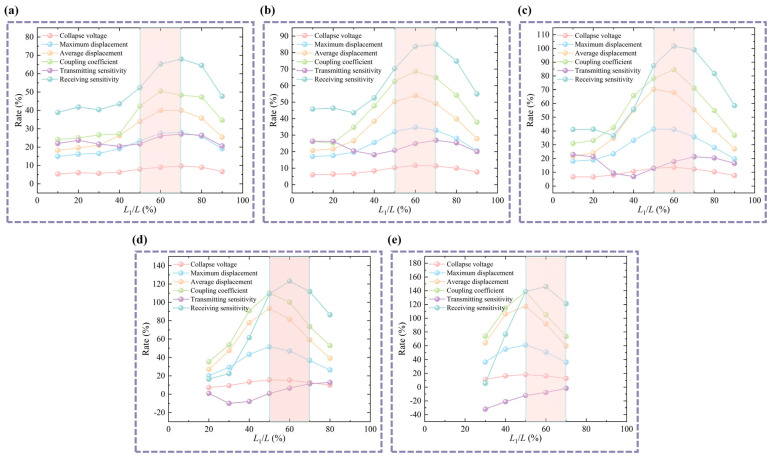
Analysis of the variation in the position of the top pillar acting on the bottom cavity. (**a**) Analysis of the variation in the position of the top pillar acting on the bottom cavity *L*_1_/*L* under *D* = 1 µm and *L*/*R* = 60%. (**b**) Analysis of the variation in the position of the top pillar acting on the bottom cavity *L*_1_/*L* under *D* = 1 µm and *L*/*R* = 80%. (**c**) Analysis of the variation in the position of the top pillar acting on the bottom cavity *L*_1_/*L* under *D* = 1 µm and *L*/*R* = 100%. (**d**) Analysis of the variation in the position of the top pillar acting on the bottom cavity *L*_1_/*L* under *D* = 1 µm and *L*/*R* = 120%. (**e**) Analysis of the variation in the position of the top pillar acting on the bottom cavity *L*_1_/*L* under *D* = 1 µm and *L*/*R* = 140%.

When the position of the top pillar acting on the bottom cavity changes, the stiffness of the top pillar and the deflection of the bottom membrane will change accordingly. Therefore, it is critical to optimize the position of the top pillar acting on the bottom cavity. To ensure this, the bottom membrane thickness (*D*) is 1 μm, the bottom cavity width ratio (*L*/*R*) and other dimensional parameters remain unchanged, and the top circular CMUT cell always matches the structural dimensions of the conventional CMUT. By adjusting the width of the middle pillar (*R*_1_) and bottom pillar (*R*_2_), the bottom cavity can be moved without changing the width of the bottom cavity, thus achieving a change in the position of the top pillar acting on the bottom cavity. In Figure 9, by analyzing the positional changes of the top pillar acting on the bottom cavity, it can be seen that most of the optimal performance occurs at *L*_1_/*L* = 50−70%. In addition, it can be seen that the optimum points of the collapse voltage reduction rate, maximum deflection improvement rate, average deflection improvement rate, electromechanical coupling coefficient rate, and receiving sensitivity improvement rate are gradually moved to the left as the bottom cavity width ratio (*L*/*R*) increases from Figure 9. Naturally, when the bottom cavity width ratio (*L*/*R*) is fixed at 120% and 140%, some positions of the top pillar acting on the bottom cavity cannot be achieved, but this does not affect the final result because the end point of the top pillar acting on the bottom cavity is usually a non-optimal point. Considering the balance between the various performances, the position of the top pillar is near *L*_1_/*L* = 60% (corresponding to the rectangular color area).

**Figure 10:**
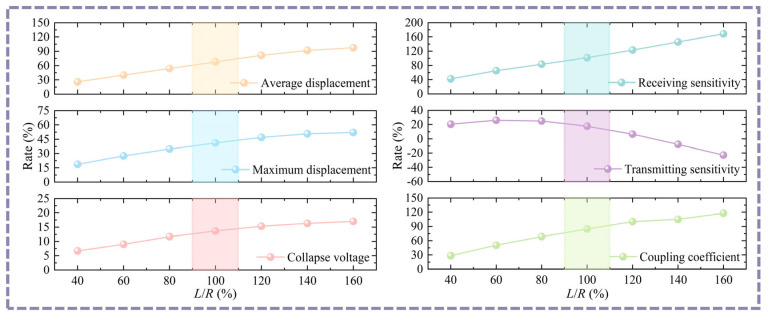
Analysis of variation in the bottom cavity width ratio *L*/*R* under *D* = 1 um and *L*_1_/*L* = 60%.

The bottom cavity width ratio will have an effect on the stiffness of the top pillar and the deflection of the bottom membrane. Therefore, the bottom cavity width ratio can be adjusted by changing the dimensions of the middle pillar (*R*_1_) and bottom pillar (*R*_2_) while keeping the bottom membrane thickness (*D*) at 1 μm, the position of the top pillar acting on the bottom cavity (*L*_1_/*L*) at 60%, and other structural parameters unchanged, and making sure that the top circular CMUT cell always matches the structural dimensions of the conventional CMUT. (It should be noted that the position of the top pillar acting on the bottom cavity (*L*_1_/*L* = 60%) remains unchanged during the adjustment of the bottom cavity width). In Figure 10, the key parameters such as collapse voltage reduction rate, maximum deflection improvement rate, average deflection improvement rate, electromechanical coupling coefficient improvement rate, and receiving sensitivity improvement rate gradually increase with the increase in the *L*/*R* ratio. However, the transmitting sensitivity improvement rate decreases as the *L*/*R* ratio increases. When the *L*/*R* ratio exceeds 100%, the decreasing rate of the transmitting sensitivity rate curve has significantly increased. In order to ensure the transmitting sensitivity performance of D-CMUTs while maximally improving their performance such as collapse voltage and receiving sensitivity, a *L*/*R* ratio of 100% (corresponding to the rectangular colored region) was chosen, resulting in a bottom cavity width of 50 µm. Naturally, for application scenarios requiring low power consumption and high receiving sensitivity, the *L*/*R* ratio can be appropriately increased.

We optimized the structure of the D-CMUT by balancing various performance factors: when the D-CMUT had *D* = 1 µm, *L*_1_/*L* = 60%, and *L*/*R* = 100% (the bottom cavity width was 50 µm), the collapse voltage reduction rate was 13.7%, the maximum deflection improvement rate was 41.2%, the average deflection improvement rate was 68.0%, the electromechanical coupling coefficient improvement rate was 84.6%, the transmitting sensitivity improvement rate was 17.7%, and the receiving sensitivity improvement rate was 101.6%.

### 4.2. Discussion

The FEM study of the D-CMUT showed that the D-CMUT not only had low power consumption, but also had high ultrasonic reception performance while maintaining transmission performance. Compared with the conventional CMUT, the D-CMUT had advantages in terms of collapse voltage and receiving sensitivity. These advantages were further enhanced by structural optimization of *D* = 1 µm, *L*_1_/*L* = 60%, and *L*/*R* = 100%. Compared with other current research (Table 3) which has been unable to achieve low power consumption and high performance at the same time, D-CMUTs show obvious advantages in combining low power consumption and high ultrasonic reception performance, providing innovative ideas and a good foundation for the improved development of CMUTs in the fields of portable, low-power devices and air-coupled ultrasonic nondestructive testing.

**Figure 11:**
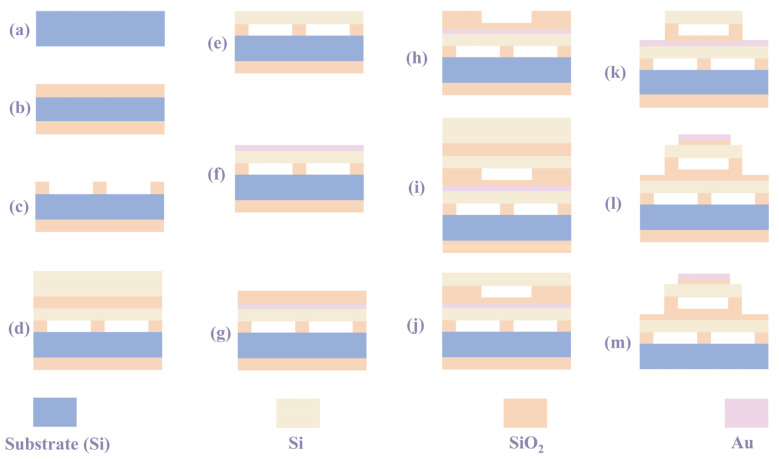
The typical fabrication process design for D-CMUTs. (**a**) Low resistance silicon wafer. (**b**) The silicon dioxide insulating layer is obtained through thermal oxidation. (**c**) Dry etching is performed on the insulating layer. (**d**) Direct bonding. (**e**) The buried silicon layer is thinned down and removed. (**f**) Sputtering of the bottom electrode. (**g**) Sputtering of the silicon dioxide insulating layer. (**h**) Dry etching is performed on the insulating layer. (**i**) Direct bonding. (**j**) The buried silicon layer is thinned down and removed. (**k**) Dry etching is performed on the silicon wafer and insulating layer. (**l**) Sputtering of the silicon dioxide insulating layer and top electrode. (**m**) The silicon dioxide insulating layer is removed.

The feasible fabrication process design of the D-CMUTs is shown in Figure 11. Although there will be an increase in the number of steps in the manufacturing process, the manufacturing process used will still be routine. Therefore, this does not affect the manufacturing feasibility of the D-CMUT. In practical applications, D-CMUTs can reduce cost by virtue of a low collapse voltage, which provides a new idea for portable, low-power devices. At the same time, the D-CMUT has a good applicability in nondestructive testing and medical imaging due to its high receiving sensitivity and other characteristics. In addition to this, the D-CMUT will have a lower frequency compared with conventional CMUTs, so it is suitable for applications in air coupling. With the continuous improvement in manufacturing process technology, the manufacturing of D-CMUTs will be increasingly stable The structural design optimization of the D-CMUT in this paper will provide a good structural parameter basis for the manufacturing of D-CMUTs.

This study of structural design optimization provides a feasible basis for future fabrication of D-CMUTs. Referring to the structural design parameters in this paper during the manufacturing process will be beneficial in obtaining the optimal performance of the D-CMUTs. In future research, the D-CMUTs can be processed and fabricated with the structural design optimization parameters provided in this paper, and the processed chips can be characterized and tested.

## 5. Conclusions

In this paper, a dual-layer CMUT is proposed, where the top cell is a circular CMUT and the bottom cell is an annular CMUT. By utilizing the deflection of the top pillar, the total deflection during operation is increased, and performance parameters such as the collapse voltage are optimized. In addition, due to the reduced stiffness of the top pillar, the D-CMUT membrane is more susceptible to deformation under external forces, resulting in higher reception performance. An FEM was built by COMSOL 6.1a. By optimizing the structural design and considering the balance between various performance factors, the D-CMUT showed excellent overall performance when *D* = 1 μm, *L*_1_/*L* = 60%, and *L*/*R* = 100%. Compared with the conventional CMUT, the collapse voltage reduction rate was 13.7%, and the improvement rates of maximum deflection, average deflection, electromechanical coupling coefficient, transmitting sensitivity, and receiving sensitivity were 41.2%, 68.0%, 84.6%, 17.7%, and 101.6%, respectively. All the data indicated that the D-CMUT achieved low power consumption and high ultrasonic reception performance while maintaining transmission performance. Therefore, this research holds great promise for applications in areas such as portable, low-power devices and cavity-coupled ultrasonic nondestructive testing.

## Figures and Tables

**Table 1:** Structural parameters and material properties of D-CMUT.

Parameter	Value	Parameter	Value
Top Si membrane radius	47.5 (µm)	Top Si membrane thickness	1 (µm)
Bottom Si membrane radius	75 (µm)	Bottom Si membrane thickness (*D*)	1 (µm)
Si substrate radius	100 (µm)	Si substrate thickness	1 (µm)
Density of Si	2332 kg/m^3^	Poisson’s ratio of Si	0.28
Young’s modulus of Si	130 GPa	Relative permittivity of Si	11.7
Top SiO_2_ pillar width	5 (µm)	Top SiO_2_ pillar height	0.5 (µm)
Bottom SiO_2_ pillar width (*R*_2_)	25 (µm)	Bottom SiO_2_ pillar height	0.5 (µm)
Middle SiO_2_ pillar radius (*R*_1_)	25 (µm)	Middle SiO_2_ pillar height	0.5 (µm)
Density of SiO_2_	2200 kg/m^3^	Poisson’s ratio of SiO_2_	0.17
Young’s modulus of SiO_2_	70 GPa	Relative permittivity of SiO_2_	4.2
Top cavity radius	47.5 (µm)	Top cavity height	0.5 (µm)
Bottom cavity width (*L*)	50 (µm)	Bottom cavity height	0.5 (µm)
Density of water	1000 kg/m^3^	The speed of sound in water	1500 m/s

**Table 2:** The fitting exponential function parameters of the device capacitance at *D* = 1 um, *L*_1_/*L* = 50%, *L*/*R* = 100%.

Formula	$C^{S}{=y}_{0}{+A}_{1}e^{\frac{{x-x}_{0}}{t_{1}}}{+A}_{2}e^{\frac{{x-x}_{0}}{t_{2}}}$		
Parameters	Numerical Value	Standard Error	Pertinence
y0	0.13874	5.2683 × 10^−5^	0.93653
x0	14.77162	--	1
A1	1.32861 × 10^−9^	--	1
t1	0.715	0.02638	0.99983
A2	0.00468	--	1
t2	7.49084	0.08986	0.98763

**Table 3:** Comparison of CMUTs with different structures.

Structures	Structure	Reduction in *V*_colla_	Increase in *w*_ave_	Increase in $K_{T}^{2}$	Increase in Receiving Sensitivity
D-CMUT	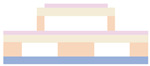	13.7%	68.0%	84.6%	101.6% (water)
Piston-shaped membranes [25]	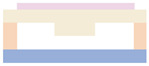	−628.1%	/	/	~100%
Annular electrodes CMUT [29]	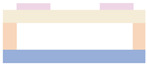	−175.1%	300%	11%	/
Annular cell geometry [28]	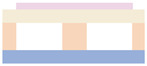	−632.8%	76.0%	/	/
High-k insulation layer [22]	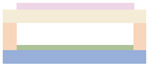	11.3%	23.7%	37.3%	49.0% (water)

## Data Availability

The original contributions presented in the study are included in the article.
